# A novel mouse calvarial defect model of osteomyelitis induced by *Staphylococcus aureus* (ATCC 6538)

**DOI:** 10.1186/s42826-026-00289-3

**Published:** 2026-09-21

**Authors:** Seongju Ko, Heung Myong Woo, Kihoon Kim, Junhyung Kim

**Affiliations:** College of Veterinary Medicine, Kangwon National University, Chuncheon- si, Gangwon-do 24341 Republic of Korea

**Keywords:** Osteomyelitis, Mouse calvarial defect, *Staphylococcus aureus*, Bone infection model

## Abstract

**Background:**

Osteomyelitis is a severe bone infection that causes chronic inflammation, tissue necrosis, and vascular compromise. The infection often requires surgical debridement and prolonged high-dose antibiotic therapy. Despite numerous osteomyelitis models in different species and skeletal sites, a standardized mouse calvarial defect model that reflects cranial intramembranous ossification and enables research on craniofacial infection is unavailable. Therefore, this study aimed to establish a reproducible mouse calvarial defect osteomyelitis model and to define an inoculum threshold that reliably induces infection while preserving experimental feasibility for downstream mechanistic and therapeutic studies.

**Results:**

Bilateral calvarial defects (diameter = 4 mm) were created in C57BL/6J mice, and the defects were inoculated with graded doses of *Staphylococcus aureus *(ATCC 6538). Four weeks postoperatively, micro-computed tomography (micro-CT), bacteriological culture, and histopathological scoring for inflammation, necrosis, and bacterial presence were performed. Mice receiving 5 µL of 10^8^ colony-forming units (CFU)/mL showed clear osteomyelitis, with significantly lower bone volume/total volume and defect union scores, together with higher CFU counts and histologic scores, compared with the controls (all *p* < 0.05). Bone mineral density showed no significant pairwise difference between groups, but a significant linear decreasing trend was detected across the ordered groups (*p* for trend = 0.0031). The lower inoculum groups exhibited only mild or no significant changes upon micro-CT imaging and histological evaluation, indicating incomplete or absent infection. Consequently, 10^8^ CFU/mL was identified as a practical cutoff for consistently inducing destructive cranial osteomyelitis in this model.

**Conclusions:**

This study established a mouse model of calvarial defect osteomyelitis that recapitulates the key features of cranial bone infection, including bone loss, impaired union, and robust inflammatory and bacterial burdens. This model provides a platform for studying the mechanisms of cranial osteomyelitis by incorporating genetically modified mice and testing local and systemic therapeutic strategies. Although only a single *S. aureus *strain and limited ancillary staining were used, the study data lay the groundwork for future investigations that incorporate diverse clinical isolates, comorbid conditions, and advanced regenerative or anti-infective interventions targeting craniofacial bone infections.

## Background

Osteomyelitis is a severe inflammatory process of bone that progresses to structural damage, most commonly caused by infection [1–3]. As osteomyelitis becomes chronic, various inflammatory mediators induce tissue necrosis, and inflammation-related impairment of the blood supply leads to ischemia, which further contributes to bone necrosis. Subsequently, necrotic tissue fragments separate to form sequestra, where antibiotic penetration is poor and often results in the failure of medical treatment [1, 4]. Therefore, successful therapy requires not only systemic and/or local administration of high-dose antibiotics but also complete surgical debridement of all necrotic tissue [5–7].

Osteomyelitis is classified into three major types according to its etiology: (1) osteomyelitis secondary to contiguous spread from adjacent infected sites, (2) osteomyelitis associated with vascular insufficiency, and (3) hematogenous osteomyelitis [1, 3]. Various animal models mimicking these pathological patterns of osteomyelitis have been developed and used in experimental research. However, no single animal model adequately represents all forms of osteomyelitis. Consequently, diverse modeling strategies have been established across multiple species, including rodents, rabbits, canines, and porcines [8–10]. *Staphylococcus aureus* is the pathogen most frequently used in animal models of osteomyelitis, as it is the predominant organism isolated from bone infections in clinical settings [4, 9, 10].

Despite the numerous advantages of mice as experimental animals—including easy handling, relatively low overall cost, feasibility of genetic manipulation, and suitability for investigating osteomyelitis-related biomarkers—mice have not been widely adopted as osteomyelitis models. This is mainly due to technical challenges related to their small size, difficulty in surgical procedures, and the limited feasibility of two-stage revision surgeries [9, 10]. Moreover, the few available mouse studies focus almost exclusively on the long bones [9, 11–13]. However, in clinical practice, osteomyelitis does not occur solely in long bones; the infection also develops in the skull [14]. The skull is formed by intramembranous ossification, which differs fundamentally from the endochondral ossification that characterizes long bones [15]. These distinct ossification mechanisms result in differences in vascularization, regenerative microenvironments, and responses to scaffolds. Hence, directly extrapolating the findings of long-bone osteomyelitis research to the skull is difficult. In addition, skull osteomyelitis is more challenging to manage than osteomyelitis of other bones owing to its complex cranial anatomy and cosmetic considerations [16].

Skull osteomyelitis has been investigated in animal models including rabbits and rats [9]. However, due to technical and modeling limitations, experimental induction of skull osteomyelitis has not yet been established in mice. Considering the unique advantages of mice over other species, these animals are likely to be valuable in osteomyelitis research. Therefore, this study designed and established a mouse calvarial defect model of osteomyelitis. Given that *S. aureus* is one of the most frequently isolated pathogens in skull osteomyelitis, the bacterium was selected as the causative organism [17].

## Methods

### Preparation of bacterial inoculum

*S. aureus* (ATCC 6538) was streaked onto tryptic soy agar (TSA) and incubated at 37 °C for 24 h. A single colony was then inoculated into 5 mL of tryptic soy broth (TSB) and cultured overnight. The bacterial density of the suspension was adjusted to a 0.5 McFarland standard (equivalent to approximately 1.5 × 10^8^ CFU/mL) using a digital densitometer (DensiCHEK Plus; BioMerieux, Marcy-l’Etoile, France). This suspension was further diluted with sterile PBS to achieve a standardized stock concentration of 10^8^ CFU/mL. Subsequently, serial 10-fold dilutions were prepared in sterile PBS to obtain concentrations of 10^5^, 10^6^, 10^7^, and 10^8^ CFU/mL. The actual concentration of each inoculum was retrospectively verified by back-plating on TSA, and the final delivered doses were confirmed to be approximately 5 × 10^2^, 5 × 10^3^, 5 × 10^4^, and 5 × 10^5^ CFU per defect (5 µL per site).

### Animal model and surgery

C57BL/6J mice (eight-week-old males, 25 g) were purchased from Dooyeol Biotec (Seoul, Republic of Korea). A total of 27 mice were randomly allocated into five groups: the control group (*n* = 3; sterile PBS, 5 µL per defect), G1 (*n* = 6; *S. aureus* (ATCC 6538) at 10^5^ CFU/mL, 5 µL per defect), G2 (*n* = 6; 10^6^ CFU/mL), G3 (*n* = 6; 10^7^ CFU/mL), and G4 (*n* = 6; 10^8^ CFU/mL). Mice were housed in an animal biosafety level 2 facility under specific pathogen-free conditions in individually ventilated cages (IVC; floor area 501 cm^2^; maximum five mice per cage) with autoclaved corn-cob bedding. The environment was maintained at 22 ± 2°C and 55 ± 10% relative humidity on a 12-h light/12-h dark cycle. Sterilized diet and purified water were provided ad libitum. Environmental enrichment, consisting of nesting materials, was provided in every cage in accordance with the institutional guidelines of the Kangwon National University Animal Laboratory Center. All protocols were approved by the Institutional Animal Care and Use Committee (IACUC) of Kangwon National University (IACUC Approval Number KW-240625-1) and were conducted at the animal biosafety level 2 facility of the Kangwon National University Animal Laboratory Center.

**Fig. 1:**
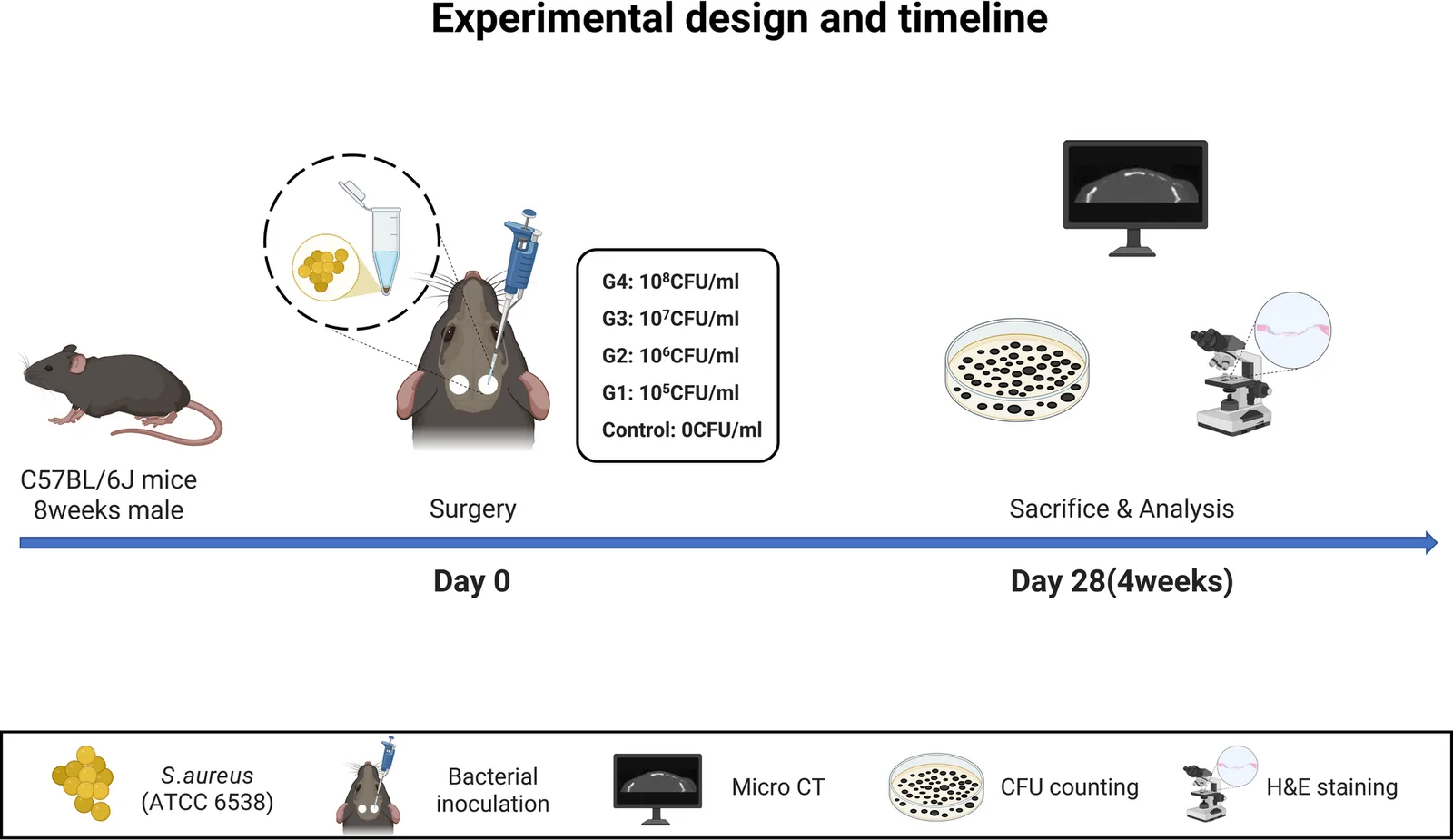
Overview of the experimental design. Mice were divided into five groups, including a control group, with different bacterial inoculum doses. Mice underwent calvarial defect surgery followed by colony-forming unit (CFU) quantification, histological staining, and micro-computed tomography (micro-CT) analysis four weeks postoperatively. Created with BioRender.com

All mice were allowed to acclimatize for one week after arrival. Anesthesia was induced by intraperitoneal injection of a combination of ketamine (80 mg/kg) and xylazine (10 mg/kg). A midline skin incision was made over the skull, followed by an incision of the periosteum, and bilateral calvarial defects were created using a 4-mm diameter trephine bur. In the control group, 5 µL of phosphate-buffered saline (PBS) was inoculated into each defect. In the four experimental groups, 5 µL of a *S. aureus* (ATCC 6538) suspension was inoculated into each defect. The following bacterial concentrations were used: group 1 (G1; 10^5^ colony-forming units [CFU]/mL), group 2 (G2; 10^6^ CFU/mL), group 3 (G3; 10^7^ CFU/mL), group 4 (G4; 10^8^ CFU/mL). Mice were euthanized with CO_2_ using the gradual-fill method four weeks after surgery. After loss of consciousness and at least 5 minutes of continued exposure, the mice were euthanized via cervical dislocation (Fig. 1).

### Micro-computed tomography analysis

Micro-computed tomography (micro-CT) scans (Quantum GX2 micro-CT, PerkinElmer, Waltham, MA, USA) were acquired for all mice at 90 kVp and 88 µA using the same scanner. The isotropic voxel size was 72 μm. After micro-CT acquisition, three-dimensional reconstruction and analyses were performed using Analyze 14.0 (AnalyzeDirect, Inc., Overland Park, KS, USA). For BMD calibration, Hounsfield units were converted to mg HA/cm³ using a linear regression curve derived from a hydroxyapatite phantom. BV/TV was quantified within a cylindrical volume of interest (VOI; 4 mm in diameter and 0.3 mm in height) centered on each defect; bone tissue was segmented using a global threshold established from the control group’s density distribution. The extent of bony bridging was semi-quantitatively scored on a 0–4 scale adapted from a previous study [18], where 4 indicated complete bridging across the entire 4-mm span of the defect; 3, bridging over a partial length; 2, bridging only at the defect borders; 1, scattered bony spicules within the defect; and 0, no new bone formation.

### Bacteriological analysis

After micro-CT scanning, the calvaria of each mouse were harvested. The right-side defect was used for CFU determination, and the left-side defect was used for hematoxylin and eosin (H&E) staining. For CFU analysis, a square calvarial segment with an 8-mm side length surrounding the defect was collected. The specimen was placed in a sterile bag and minced with sterile scissors, followed by the addition of 10 mL of PBS. The samples were then processed in an ultrasonic homogenizer for 30 s, allowed to rest, and sonicated for 30 s. After centrifugation, the supernatant was discarded, and the pellet was resuspended in PBS (0.5 mL). A 0.3 mL aliquot was plated on Baird-Parker agar and spread evenly before incubation. The CFUs were counted after 48 h of incubation.

### Histological analysis

After removing the bone used for CFU measurements, the remaining specimens were fixed in 10% neutral buffered formalin solution. Fixed specimens were washed, decalcified overnight, and embedded in paraffin. The sections were cut to a thickness of 3 μm and stained with H&E. The inflammation score, bone necrosis score, and presence of bacteria were evaluated using a previously published semi-quantitative scoring system, and the sum of these parameters was used as the composite histological score [19, 20]. Histological sections were evaluated in a blinded manner by two independent observers.

### Statistical analyses

All micro-CT outcomes were derived from the same scans but analyzed according to their measurement scale. For BMD and BV/TV, nested one-way ANOVA with Tukey’s multiple comparisons test was performed, with each mouse modelled as a random effect to account for the bilateral design. Assumptions of residual normality and homoscedasticity for the nested ANOVA were verified by inspection of QQ plots, residual-versus-predicted plots, and homoscedasticity plots. To assess the dose-response relationship across ordered groups (Control → G1 → G2 → G3 → G4), a test for linear trend was additionally performed on per-animal averaged values.

Bone bridging score, a semi-quantitative ordinal variable derived from micro-CT reconstruction, was analyzed using nonparametric methods. To account for the bilateral design, scores from the two defects were averaged per mouse prior to analysis. Group differences were assessed using the Kruskal–Wallis test with Dunn’s post hoc test, and a Jonckheere–Terpstra test was performed to evaluate ordered trends across groups.

CFU counts were log-transformed prior to analysis and compared across groups using the Kruskal–Wallis test with Dunn’s post hoc test. Histological outcomes (inflammation, bone necrosis, presence of bacteria, and composite score) were analyzed using the same nonparametric approach.

All statistical analyses were performed using GraphPad Prism version 11.0.0 (GraphPad Software Inc., San Diego, CA, USA), with the exception of the Jonckheere–Terpstra trend test, which was conducted in Python using a custom implementation based on the normal approximation with ties correction (Python 3.12; NumPy 2.4.3; SciPy 1.17.1). Quantitative continuous variables (BMD and BV/TV) are expressed as mean ± standard deviation (SD). For ordinal scoring data (bone bridging and histological scores) and non-normally distributed continuous data (CFU counts), the results are presented as median with interquartile range (IQR).

## Results

### Clinical observations

All mice survived until the scheduled four-week endpoint, with no mortality in any group. Throughout the study period, no animals exhibited clinical signs of systemic illness, such as hunched posture, piloerection, reduced activity, or decreased food/water intake.

### Micro-CT analysis

**Fig. 2:**
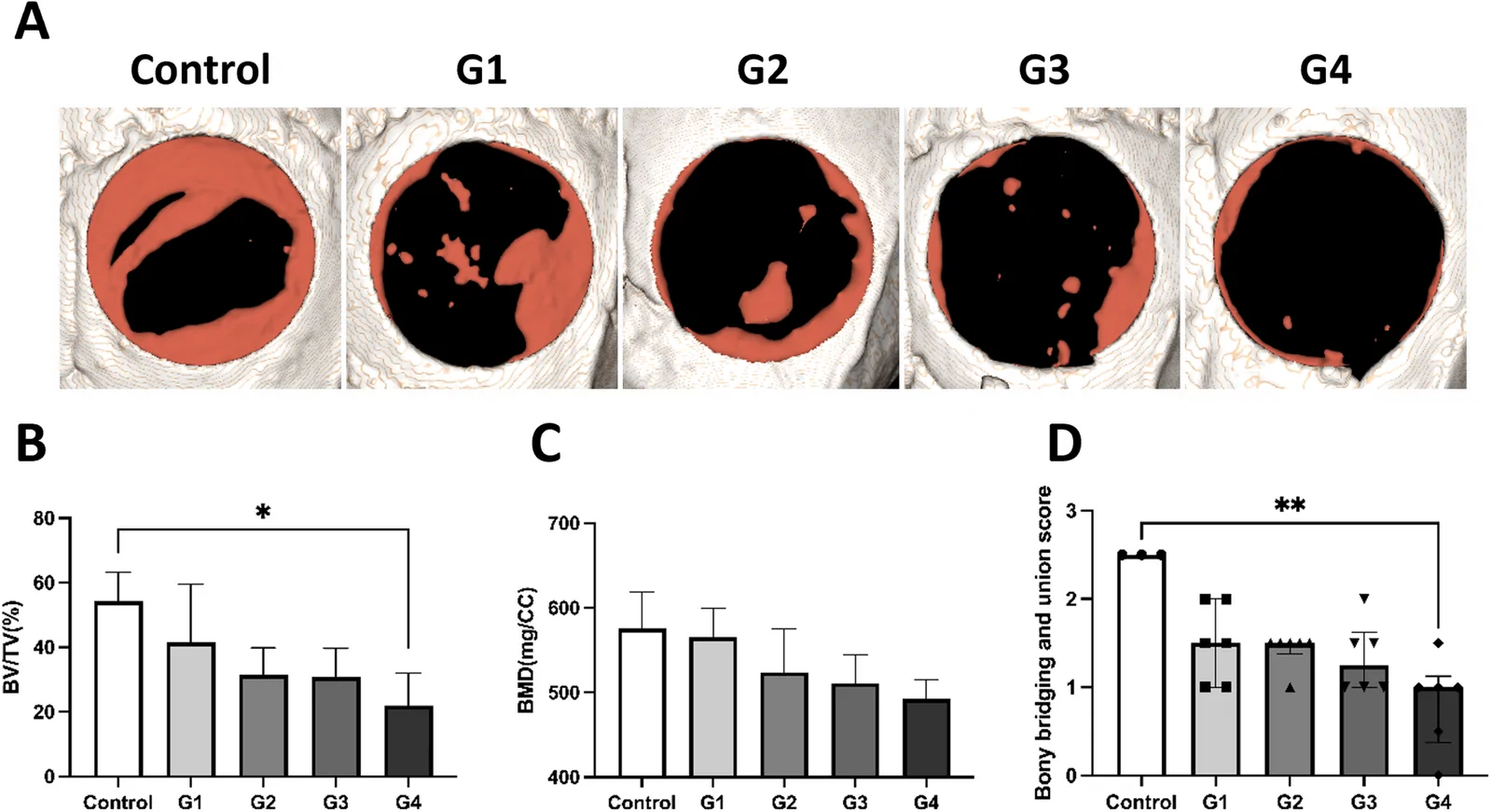
(**A**) Representative 3D micro-CT reconstruction images for each group. (**B**) Bone volume/total volume (BV/TV), (**C**) bone mineral density (BMD), and (**D**) bony bridging and union scores. Group G4 showed significantly lower values compared to the control group in BV/TV and bony bridging and union scores. Data in (**B**) and (**C**) are presented as mean ± SD, while data in (**D**) are expressed as median with interquartile range (IQR). *p < 0.05; **p < 0.01

On micro-CT reconstruction images (Fig. 2A), higher bacterial inoculum levels were associated with reduced bone regeneration. In terms of BV/TV (Fig. 2B), group G4 showed significantly lower values than those of the control and G1 groups (*p* < 0.05), and a significant linear trend was observed across groups with increasing inoculum concentration (*p* = 0.0003). Although no significant differences were found between groups in BMD (Fig. 2C), a significant linear trend was similarly identified (*p* = 0.0031), suggesting a progressive decrease in BMD with higher bacterial inoculum levels. Bony bridging and union scores were significantly lower in group G4 than in the control group (*p* < 0.01) (Fig. 2D). No significant differences were observed between the control group and groups G1, G2, and G3 for any of these parameters.

### Bacteriological analysis

**Fig. 3:**
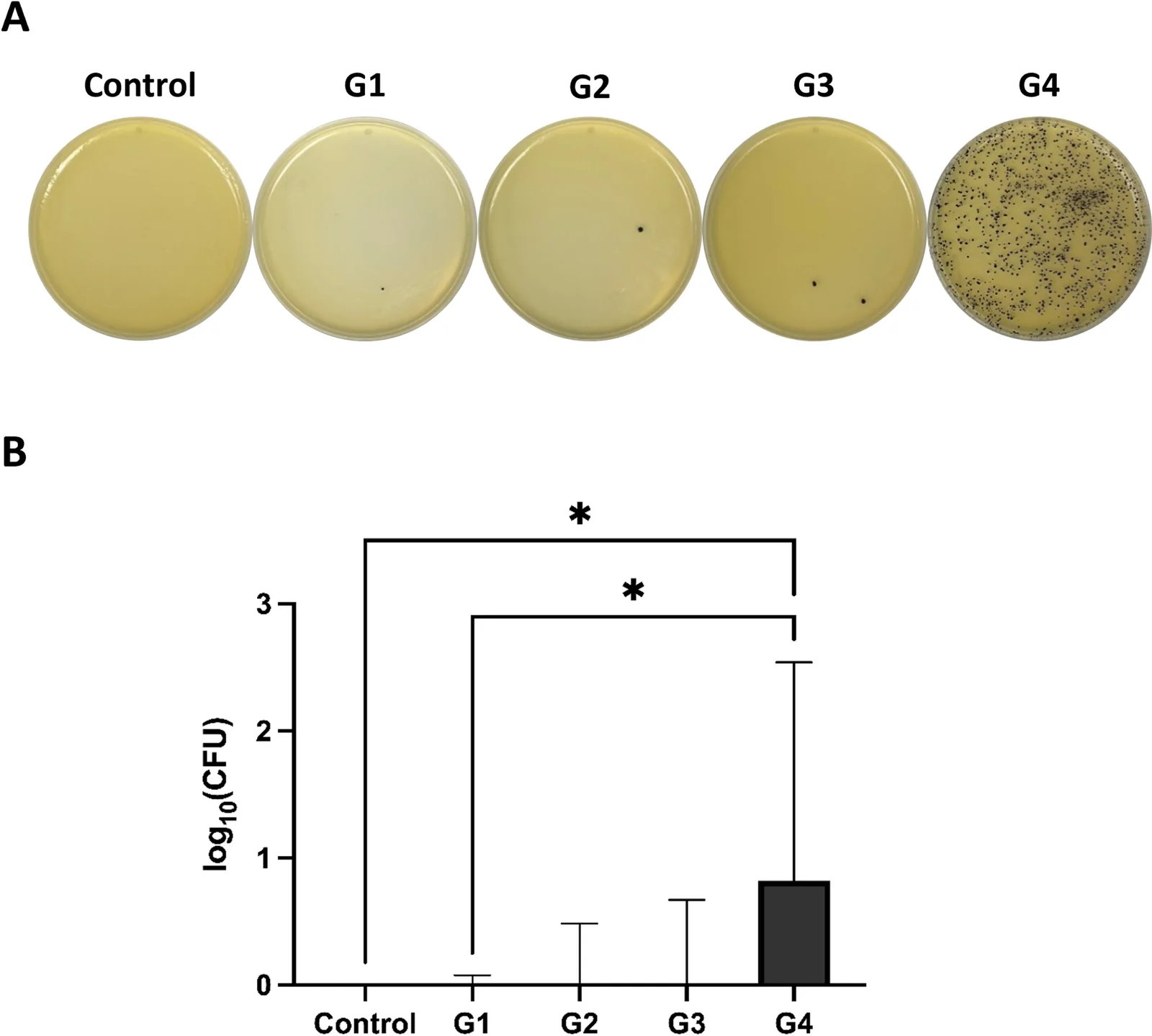
(**A**) Representative bacterial colonies cultured from bone samples. (**B**) Comparison of mean colony-forming unit (CFU) counts per group showed significantly higher values in group G4 than in the control and G1 groups. *p < 0.05

The mean bacterial CFUs recovered from homogenized calvarial bone surrounding each defect were as follows: Control, 0 CFU; G1, 1.7 × 10^− 1^ CFU; G2, 1.8 × 10⁰ CFU; G3, 3.2 × 10^0^ CFU; and G4, 2.77 × 10² CFU (Fig. 3A). In group G4, the number of cultured bacteria was significantly higher than that in the control and G1 groups (*p* *< 0.05*) (Fig. 3B). Although bacterial growth was observed in groups G1, G2, and G3, not all samples were culture-positive, and no statistically significant differences were observed compared with the control group.

### Histological analysis

**Fig. 4:**
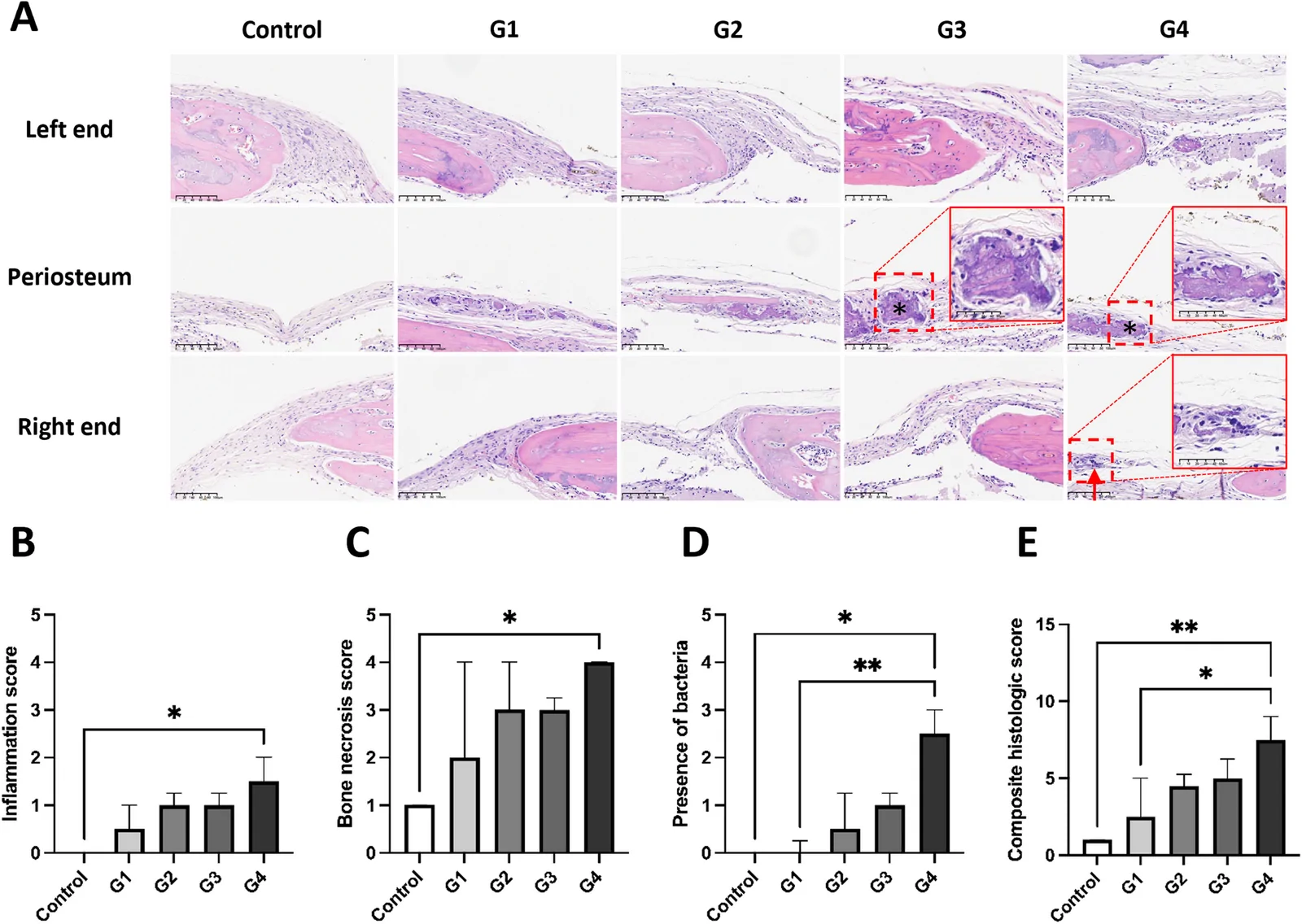
(**A**) Hematoxylin and eosin (H&E) staining of the defect margins and periosteum in each group. In group G4, sequestra and bacterial colonies were observed. Group G4 showed significantly higher inflammation (**B**), bone necrosis (**C**), presence of bacteria (**D**), and composite histologic scores (**E**) than those of the control group. Scale bar = 100 μm; inset scale bar = 50 μm. Asterisk, sequestrum; red arrow, bacterial colonies. *p < 0.05; **p < 0.01

Calvarial defects in all groups were covered by connective tissue. Even though they were left untreated, none of the mice in the control group achieved complete healing because the defects were of critical size. No inflammation or bacteria were observed in the control group, whereas groups G1, G2, and G3 showed mild inflammation with focal necrosis and a small number of bacterial colonies. In contrast, group G4 exhibited severe inflammation and bone necrosis, accompanied by numerous bacterial colonies (Fig. 4A). Group G4 exhibited a significantly higher inflammation score than that of the control group (*p* < 0.05) (Fig. 4B). Although the mean scores of groups G1, G2, and G3 were higher than those of the control group, the difference was not statistically significant. For the bone necrosis score, only group G4 showed a significantly higher score than that of the control group (*p* < 0.05) (Fig. 4C). In terms of the presence of bacteria, group G4 had a significantly higher score than that of the control (*p* < 0.05) and G1 (*p* < 0.01) groups (Fig. 4D). When the three parameters were summed to obtain the composite histologic score (Fig. 4E), group G4 showed a significantly higher score than that of the control (*p* < 0.01) and G1 (*p* < 0.05) groups. All histological scores were significantly higher in group G4 than in the control group.

## Discussion

Although osteomyelitis symptoms have been recognized for many years, the term “osteomyelitis” was first introduced by Nelaton in 1844 [21]. Osteomyelitis is commonly classified into the following three types according to its etiology: osteomyelitis secondary to contiguous infection, osteomyelitis associated with vascular insufficiency, and hematogenous osteomyelitis [1, 3]. In 1884, Rodet developed the first animal model of osteomyelitis. Since then, numerous animal models have been established [22, 23]. Osteomyelitis is not confined to a single cause or a single anatomic location. Given that many variables, such as the underlying cause, structural characteristics of the affected bone, and comorbidities, can influence the disease, establishing a single animal model to represent all forms of osteomyelitis is impossible. Therefore, a wide variety of species and skeletal sites have been used as animal models for osteomyelitis research.

The most frequently used species are rabbits, rats, and mice, which have advantages over large animals in terms of easier handling and lower costs. Animal models have been applied not only to long bones, such as the femur, tibia, and radius, which are commonly used in osteomyelitis research, but also to the skull. However, cranial osteomyelitis can arise from various causes and remains a great challenge despite advances in new antibiotics and surgical techniques [16, 24]. Most animal model studies of skull osteomyelitis have focused on inducing infection in calvarial defects and subsequently evaluating treatments [20, 25–27]. However, owing to the technical difficulty of surgery, the challenges of reliably inducing osteomyelitis, and relatively less interest in skull osteomyelitis than in long-bone osteomyelitis, no studies have established osteomyelitis in mouse calvarial defect models.

Accordingly, this study focused on the advantages of mice. These advantages included ease of manipulation, cost-effectiveness, and the ability to use transgenic and knockout mice to investigate the responses of specific genes and pathways to disease conditions. Based on these strengths, this study hypothesized that a mouse calvarial defect osteomyelitis model could effectively elucidate the mechanisms of cranial osteomyelitis and aid in developing new therapies. Hence, the study established such a model. The study aimed to determine the minimum bacterial concentration required to induce infection, by inoculating *S. aureus* at several concentrations and analyzing the outcomes four weeks after surgery.

The results showed that group G4 (10^8^ CFU/mL) exhibited pronounced differences compared with that of the control group. On the micro-CT images, a clear visual difference in the defect area was evident between the control and G4 groups. Both the planar parameter (bony bridging and union score) and the three-dimensional parameter (BV/TV) were markedly lower in group G4 (Fig. 2). When osteomyelitis develops, disruption of the receptor activator of nuclear factor kappa-B ligand (RANKL)/osteoprotegerin (OPG) balance leads to an increased RANKL/OPG ratio, enhanced osteoclast differentiation and activity, and subsequent reduction in BMD [28, 29]. Consistent with this mechanism, BMD showed a significant linear decreasing trend across the ordered groups, although no significant pairwise difference was detected between groups, suggesting a progressive reduction in mineralization with increasing bacterial inoculum. Bacterial cultures of harvested bone samples also demonstrated markedly higher CFU counts in group G4 (Fig. 3). For an objective histological evaluation, a semi-quantitative scoring system was applied for inflammation, bone necrosis, and bacterial presence as described in previous studies [19, 20]. The scores tended to increase at higher inoculum concentrations and the group G4 score was considerably higher than that of the control group (Fig. 4). Collectively, these findings confirmed that osteomyelitis was successfully induced in group G4. This also aided in determining the cut-off concentration of the *S. aureus* suspension required to induce calvarial defect osteomyelitis in mice.

Taken together, these findings support the successful establishment of a mouse calvarial defect osteomyelitis model. Given the heterogeneity of osteomyelitis in terms of etiology, anatomical location, and host factors, no single animal model can encompass all disease manifestations. Among the available options, small animals such as rabbits and rats have been widely adopted for their practicality, and calvarial defect models in these species have been used to evaluate treatment strategies. However, no equivalent model had been established in mice, despite the distinctive advantages the mouse platform offers—including cost-effectiveness, ease of handling, and compatibility with transgenic and knockout approaches. The present study addresses this gap by defining a minimum inoculum concentration of *S. aureus* sufficient to reproducibly induce osteomyelitis in mouse calvarial defects.

Although this study successfully induced osteomyelitis in mice with calvarial defects, some limitations should be acknowledged. First, no additional staining was performed beyond H&E staining. Although some studies rely solely on H&E, the use of additional stains, such as osteocalcin, or immunohistochemical markers, such as cyclooxygenase-2, could have provided more precise histological assessment of bone regeneration and inflammation. Nevertheless, given that micro-CT and bacteriological analyses were performed, the lack of additional staining does not substantially undermine the reliability of the results. Second, only one *S. aureus* strain (ATCC 6538) was used. Considering that virulence factors, biofilm-forming capacity, and host responses can vary, even within the same species, the cut-off concentration determined in this study cannot be generalized to all *S. aureus* strains. Nevertheless, the study findings provide useful guidelines for determining inoculum concentrations for future studies aimed at inducing and treating calvarial defect osteomyelitis in mice.

Despite these limitations, the study is important in that it demonstrates the reproducible induction of bone infection in mouse calvarial defects. Accordingly, further osteomyelitis studies using gene-mutant mice with calvarial defects may help clarify changes in signaling pathways and immune responses in the osteomyelitis microenvironment and contribute to the development of novel therapeutic strategies. Cranial osteomyelitis is known to occur more readily in patients with underlying conditions, such as advanced age, diabetes, and immunosuppressive diseases. Therefore, by establishing combined models in which osteomyelitis is induced in the presence of such comorbidities, future work may enable the investigation of treatment strategies for osteomyelitis under more challenging clinical conditions.

## Conclusions

This study established a novel mouse calvarial defect model of osteomyelitis by inoculating bilateral skull defects (4-mm diameter) in C57BL/6J mice with graded concentrations of *S. aureus* (ATCC 6538). Overall, 5 µL of 10⁸ CFU/mL induced destructive cranial osteomyelitis, as demonstrated by micro-CT, bacteriological, and histological evidence of severe bone loss and infection compared with that of controls. In contrast, lower bacterial concentrations produced only mild or inconsistent changes. These data define a practical cut-off inoculum to reliably induce skull osteomyelitis in mice and indicate that this model captures key features of cranial bone infection. This provides a versatile platform for studying disease mechanisms in transgenic or knockout mice and preclinical evaluation of local and systemic anti-infective or regenerative therapies tailored to the craniofacial bone. Although the current study used only a single *S. aureus* strain, the model can be readily expanded to include additional clinical isolates, immunohistochemical and molecular analyses, and comorbidity models to enhance its translational relevance in complex cranial osteomyelitis.

## Data Availability

All data generated or analyzed in this study are available from the corresponding author upon reasonable request.

## Abbreviations

BV/TV: Bone volume / total volume
BMD: Bone mineral density
CFU: Colony-forming units
G1–4: Groups 1–4
H&E: Hematoxylin and eosin
IACUC: Institutional Animal Care and Use Committee
micro-CT: microcomputed tomography
OPG: Osteoprotegerin
PBS: Phosphate-buffered saline
RANKL: Receptor activator of nuclear factor kappa-B ligand
